# High-affinity antibodies specific to the core region of the tau protein exhibit diagnostic and therapeutic potential for Alzheimer’s disease

**DOI:** 10.1186/s13195-024-01561-1

**Published:** 2024-10-02

**Authors:** Mohammad Arastoo, Lewis K. Penny, Richard Lofthouse, Aya Abdallah, Anna Abrahamsson, Pietro Marini, Valeria Melis, Gernot Riedel, Charles R. Harrington, Claude M. Wischik, Andrew Porter, Soumya Palliyil

**Affiliations:** 1 Institute of Medical Sciences, University of Aberdeen, Aberdeen, UK; 2 Scottish Biologics Facility, University of Aberdeen, Aberdeen, UK; 3 GT Diagnostics (UK) Ltd, Aberdeen, UK; 4 TauRx Therapeutics Ltd, Aberdeen, UK

**Keywords:** Alzheimer’s disease, Tauopathy, Tau protein, Paired helical filament, Monoclonal antibody, Diagnostic, Therapy, Immunotherapy

## Abstract

**Background:**

Recent advances in blood-based biomarker discovery are paving the way for simpler, more accessible diagnostic tools that can detect early signs of Alzheimer’s disease (AD). Recent successes in the development of amyloid-targeting immunotherapy approaches mark an important advancement in providing new options for the treatment of AD. We have developed a set of high-affinity monoclonal antibodies (mAbs) to tau protein that have the potential as tools for diagnosis and treatment of AD.

**Methods:**

Sheep were immunised with either full-length tau (1-441) or truncated paired helical filament (PHF)-core tau (297–391). A stringent bio-panning and epitope selection strategy, with a particular focus directed to epitopes within the disease-relevant PHF-core tau, was used to identify single-chain antibodies (scAbs). These scAbs were ranked by affinity for each epitope class, with leads converted to high-affinity mAbs. These antibodies and their potential utility were assessed by their performance in tau immunoassays, as well as their ability to prevent tau aggregation and propagation. Further characterisation of these antibodies was performed by immunohistochemical staining of brain sections and immuno-gold electronmicroscopy of isolated PHFs.

**Results:**

Our work resulted in a set of high-affinity antibodies reacting with multiple epitopes spanning the entire tau protein molecule. The tau antibodies directed against the core tau unit of the PHF inhibited pathological aggregation and seeding using several biochemical and cell assay systems. Through staining of brain sections and PHFs, the panel of antibodies revealed which tau epitopes were available, truncated, or occluded. In addition, highly sensitive immunoassays were developed with the ability to distinguish between and quantify various tau fragments.

**Conclusion:**

This article introduces an alternative immunodiagnostic approach based on the concept of a “tauosome” – the diverse set of tau fragments present within biological fluids. The development of an antibody panel that can distinguish a range of different tau fragments provides the basis for a novel approach to potential diagnosis and monitoring of disease progression. Our results further support the notion that tau immunotherapy targeting the PHF-core needs to combine appropriate selection of both the target epitope and antibody affinity to optimise therapeutic potential.

**Supplementary Information:**

The online version contains supplementary material available at 10.1186/s13195-024-01561-1.

## Introduction

### Tau protein and Alzheimer’s disease

Tau is a microtubule-associated protein that promotes their assembly and stability which in turn allows reorganisation of the cytoskeleton and subsequent neurite growth [8, 20]. Besides regulating the dynamics of microtubules, tau also regulates anterograde and retrograde axonal transport by influencing the function of the motor proteins dynein and kinesin [17].

Neurofibrillary tangles (NFTs) consisting of filamentous aggregates of tau protein are a pathological hallmark of Alzheimer’s disease (AD) [53]. Structurally, tau is an intrinsically disordered protein under normal physiological conditions, lacking significant tertiary structure [30]. However, under pathophysiological conditions, tau protein undergoes a cascade of misfolding and oligomerisation that induces neurodegeneration [33, 61]. This pathologic aggregation cascade is typically a slow process that drives the conversion of normal monomeric tau protein into small, initially soluble, oligomer fragments that subsequently over time form large, insoluble paired helical filaments (PHFs) and ultimately NFTs. This progression results eventually in the almost complete redistribution of the brain pool of tau protein from normal soluble to pathologically aggregated states [38].

The mechanisms that drive this redistribution of tau protein is not fully understood, but the substantially higher affinity of pathological tau binding to the PHF-core domain, when compared with its physiological binding to tubulin, may contribute [32]. N- and C-terminal truncation of tau protein [29] exposes residues within the core of the molecule that allow them to capture normal tau in a self-propagating cascade which accelerates the aggregation process [16, 58]. The spatiotemporal spread of tau pathology strongly correlates with neuronal loss and the cognitive decline associated with AD [6, 16, 23, 26, 39].

### Tau – biomarkers for AD

It is estimated that 50% of the population over the age of 47 have identifiable tau pathology at Braak stage 2 or beyond [60]. Tau biomarkers in biological matrices such as cerebrospinal fluid (CSF) and blood are being studied to provide a predictive window into the early diagnosis of AD [45]. Assays that measure phosphorylated tau (ptau181, ptau217 and ptau231) have shown promise in discriminating individuals on the AD continuum, even before symptoms appear. These are not used as stand-alone diagnostic tools since the relatively small changes in ptau protein require large sample numbers to demonstrate statistical significance [5, 28]. Plasma ptau217 has proved the best-in-class to date as a singular tau biomarker of AD biology [48, 57].

There are a number of paired antibody assays that claim to be able to quantify levels of ‘total’ tau [63]. Whilst robust in their analytical performance, detecting all isoforms of tau, it is clear from our growing understanding of the tauosome that these assays do not measure all tau species because the antibody pairings lack the ability to detect certain truncated tau fragments, and especially those which contain the microtubule-binding region (MTBR) core [4, 37]. Since these core tau species are heavily implicated in several tauopathies, the lack of specific, high-affinity antibodies recognising this region has hampered progress in both AD diagnostics and antibody-based therapeutics.

### Tau – a therapeutic target for AD

Several tau-based therapies for AD are now in active clinical development including antibody, vaccine, genetic and small molecule-based approaches [14]. Due to the clinical success of immunotherapy in severely debilitating disease areas such as cancer and autoimmune disorders, antibody-based approaches present a potentially attractive therapeutic option for AD. The primary purpose of anti-tau approaches has been to inhibit the oligomeric tau aggregation seeding species in the extracellular space from propagating between neurons in a prion-like manner, preventing the spread of pathology to healthy neighbouring neurons [22].

A number of tau-directed antibodies, such as semorinemab, tilavonemab, zagotenemab and gosuranemab, have all failed to show efficacy in phase 2 AD clinical trials [40]. However, given that tau is heavily truncated in AD [4, 59] and that these four antibodies bind the N-terminus of tau, the failure can be attributed at least in part to targeting epitopes that are not involved in initiating seeding pathology [15, 55]. Therefore, an attractive way forward would be to develop immunotherapeutic approaches which target the core tau domain that is critical for the aggregation cascade.

## Summary

In this report, we describe the generation of antibodies targeting core tau. The antibodies were characterised using peptide mapping, epitope binning and scanning mutagenesis, followed by affinity ranking by ELISA and surface plasmon resonance. This panel of antibodies was used to develop sensitive sandwich ELISAs that can distinguish between a range of tau fragments in the tauosome. In addition, the therapeutic potency of these antibodies was ranked *via* a cellular assay to assess anti-tau propagation activity. This study highlights the complex molecular diversity of the tau core region and provides important evidence of core-tau antibodies having both therapeutic and diagnostic potential in AD.

## Materials and methods

### Generation of anti-tau antibodies from immunised phage display libraries

Full-length tau protein (referred to as 2N4R), dGAE (PHF-core domain of 2N4R tau from amino acids 297–391) and dGA (tau297-390) proteins were expressed in bacteria and purified as previously described [1]. For sheep immunisation, 2N4R tau and dGAE were used as antigens with a primary inoculum (250 µg) followed by 4 subsequent boosts (125 µg)(Ig Innovations Ltd., Wales, United Kingdom). After confirming a high antibody titre, phage display antibody libraries capturing the immune repertoire of sheep were constructed as per published methods [11, 46]. Both 2N4R tau and dGAE libraries were subjected to stringent bio-panning and forced epitope selection strategies to drive selection towards high-affinity antibodies with rich epitope diversity (Supplementary Tables S1 and S2). Positive phage ‘hits’ were sequenced via capillary array electrophoresis and clones having unique sequences were cloned into a bacterial expression vector, pIMS147, for reformatting into single chain antibodies (scAbs) according to previously published methods [25]. A selected panel of tau scAbs were further reformatted into sheep-mouse chimeric mAbs by linking the sheep variable domains with the constant regions of mouse IgG2a isotype and expressed in a transient human embryonic kidney suspension system (HEK-293F) [46].

### Affinity ranking of tau antibody clones (ELISA)

For affinity ranking of scAbs and mAbs, MaxiSorp flat-bottom 96-well plates were coated with dGAE or 2N4R tau (1 µg/mL) and blocked with 2% (w/v) dried milk powder in PBS, prior to adding scAbs or mAbs in doubling dilution. The binding was detected using HRP-conjugated anti-human kappa light chain antibody (for scAbs; Sigma, #A7164) and HRP-conjugated anti-mouse IgG (for mAbs; Sigma, #A6782) as the secondary antibody and following standard ELISA procedure. Prism version 9.5.1 (GraphPad Software Inc., San Diego, USA) was used to fit non-linear 4-parameter logistic regression models for each scAb or mAb and 50% bound values were used to rank the immunoreactivity of clones.

### Kinetics of binding and affinity determination via surface plasmon resonance (SPR)

All SPR experiments were carried out using a Biacore X100 and performed in HBS EP + running buffer (GE, #BR-1006-69) at 25 °C. Binding responses were obtained by subtracting reference flow cell responses from the active flow cells. The active flow cell of a CM5 chip was immobilised with approximately 200 RU (response units) of 2N4R tau, using a primary amine-coupling kit (GE, #BR-1000-50). All steps were performed at a flow rate of 10 µL/min. scAbs or mAbs were injected in increasing concentrations (0.39 nM − 50 nM, minimum of 5 concentrations) at a rate of 30 µL/min across the chip. Each cycle consisted of a 120 s association phase followed by a 600 s dissociation phase and a subsequent regeneration step with 30 s injection of 10 mM glycine pH 1.5 (GE, #BR-1003-54). Results were analysed using Biacore X100 evaluation software and data fitted to a 1:1 binding model to obtain kinetic rates and equilibrium binding constants.

### Epitope determination of antibody clones using biotinylated tau peptides

MaxiSorp flat-bottom 96-well plates were coated with streptavidin (5 µg/mL; Invitrogen, #434302) and blocked as above. N-terminally biotinylated tau peptide (13 AA) of interest was added to the plate at 2 µg/mL, allowed to bind and 10 µg/mL scAb was added following washing. The scAb binding was detected and measured as described previously. For each core-tau antibody, the epitope was further characterised using a series of N-terminally biotinylated tau peptides (13 AA) with a single alanine substitution of each residue and performed as above.

### Developing ultra-sensitive diagnostic pairs for the interrogation of mixed tau species (“tauosome”)

#### Determination of lower limits of quantification (LLOQ) for ELISAs with paired mAb/scAb

For a sandwich colourimetric ELISA, 96-well plates were coated with a capture mAb at 1 µg/mL, blocked and doubling dilutions of 2N4R tau from a 1 µg/mL starting concentration were added to designated wells. A series of detection scAbs recognising various tau epitopes were added at 10 µg/mL and detected using HRP-conjugated anti-human kappa light chain antibody as described above. LLOQ was calculated as the lowest dilution in the standard curve with signal higher than the average blank value plus nine standard deviations.

#### Determination of LLOQ for chemiluminescent ELISAs with paired mAbs

A black MaxiSorp flat-bottom 96-well plate (Fisher #10030581) was coated with 2.5 µg/mL of capture mAb and blocked using phosphate-buffered saline plus nonfat milk (2%), as previously described. 2N4R tau was double diluted in triplicate from 8 to 0.125 ng/mL and added to the wells; plates were washed and various HRP conjugated mAb detector antibodies were added. Detector mAbs were directly conjugated with HRP detector using an HRP Conjugation Kit – Lightning link® (Abcam, #ab102890). Following incubation, the plates were washed 5 times in PBST before adding SuperSignal ELISA Femto Substrate (Thermo Scientific, #37075). Total luminescence was read using a Clariostar Plus microplate reader (BMG Labtech). LLOQ was calculated as described above.

#### Interrogation of mixed tau samples and their quantification using different antibody combinations

Spiked samples were prepared with varying concentrations and types of tau species: sample A with 5 nM full-length human tau (2N4R tau); sample B with 3.3 nM dGA + 3.3 nM dGAE + 3.3 nM 2N4R tau (9.9 nM total protein); sample C with 2 nM dGA; and sample D with 1 nM 2N4R tau + 4 nM dGA (5 nM total protein). Samples were analysed by performing three separate ‘blinded’ sandwich ELISAs using S1D12 mAb to capture the different species in the mixture and detected using scAbs with specific epitopes. These assays were performed similar to the previously described colorimetric mAb capture and scAb detection ELISAs. Based on ELISA signals of unknown spiked samples, the types of tau fragments present in the mixture and their individual concentrations were determined against standard curves for each tau species.

### Assessment of tau aggregation inhibition properties of scAbs

#### Thioflavin T aggregation inhibition assay

Assembly of aggregated dGAE filaments was performed on a ThermoMixer C Block (Eppendorf) by incubation of 10 µM scAb + 100 µM dGAE + 10 mM DTT for 24 h in PBS (pH 7.4) at 37 °C with 700 rpm. After 24 h, samples were loaded onto a Nunc Delta-Treated 96-well flat-bottom black plate (Thermo Scientific, #137101) with a final concentration of 12.5 µM thioflavin T (Sigma, #T3516). Fluorescence was measured with a constant emission wavelength of 480 nm and a scanning excitation wavelength of 350–470 nm using a Varian Cary Eclipse fluorescence spectrophotometer. Peak fluorescence measurements were used to quantify aggregation (450 nm excitation) after subtraction of a blank control containing 10 µM scAb + 100 µM dGAE + 10 mM DTT (without agitation). Tau scAbs were ranked according to extent of aggregation of dGAE in the presence of 10 µM scAb and compared to dGAE aggregated in the presence of a negative control scAb.

#### Tau-tau binding inhibition assay

A 96-well flat-bottom Maxisorp plate was coated with dGA (1 µM), washed and blocked as before. Doubling dilutions of scAbs were incubated with 100 nM of dGAE in binding buffer (25 mM PIPES, 50 mM NaCl, 0.05% Tween^®^ 20, 1% fish skin gelatine; pH 6.8) overnight at 4 °C on a separate polypropylene plate (Thermo Scientific, #249946). After 3 washes with PBST, scAb-dGAE complexes were added to the blocked immuno-plate and incubated for 1 h at 37 °C. Following 3 washes, mAb 423 (tau391E-specific antibody, 1:250, [43] was added to the plate and the binding was detected using HRP-conjugated anti-mouse IgG as previously described. Prism version 9.5.1 (GraphPad Software Inc., San Diego, USA) was used to fit non-linear 4-parameter logistic regression models for each scAb and 50% bound values were used to rank aggregation inhibition properties of the panel of scAbs.

### Preparation of murine and human brain homogenates

#### Line 66 (L66) tau transgenic mouse brains

A plasmid construct containing 2N4R tau carrying a double mutation, P301S and G335D, was created using PCR-directed mutagenesis. P301S is a mutation associated with frontotemporal dementia [7]. The cDNA was inserted into the murine Thy-1 expression cassette (pTSC21k) to generate L66 mice ensuring neuronal expression. These mice show sensorimotor impairments and motor learning phenotypes with abundant tau pathology expressed in neurons throughout the brain [36]. Brain homogenates for anti-tau propagation studies were derived from combining the brains from three individual 5-month-old L66 mice.

#### Human brain tissue

Prefrontal cortex (grey matter) tissue corresponding to Brodmann areas 8–10 was received from South West Dementia Brain Bank under project code MTA147 from three brain bank donors with a clinical and histological diagnosis of AD at Braak stage V/VI. Given the heterogeneity of AD and the limited numbers, a combined brain homogenate for anti-tau propagation studies was prepared from the three donors.

Brain homogenates were prepared in 1:10 (w/v) TBS with 1 x Halt Protease and Phosphatase Inhibitor Cocktail (Thermo Scientific, #78442). Homogenisation was performed with 30 strokes using a Caframo Ultra Torque (BDC1850) with pestle attachment at 200 rpm. Samples were left on ice for 1 h and subsequently centrifuged for 10 min at 10,000 x g at 4 °C. The supernatant was collected, and the total protein was quantified using the BCA Protein Assay Kit (Pierce, #23225). For Tau RD P301S FRET Biosensor Cell experiments, an aliquot of brain homogenate was taken prior to centrifugation and sonicated (6 × 10 s cycles) at 60% amplitude using a 0.5-mm probe. Samples were subsequently processed as above.

### Assessment of anti-tau propagation properties of the monoclonal antibody panel

#### Tau RD P301S FRET biosensor cell maintenance

Tau RD P301S FRET Cells (#CRL-3275) were incubated at 37 °C / 5% CO_2_ with high relative humidity and handled in a Class II cell culture laminar flow hood. Cells were maintained in high glucose (4.5 g/L) Dulbecco’s Modified Eagle Medium supplemented with 2 mM L-alanyl-L-glutamine dipeptide (GlutaMax™), 50 I.U./mL penicillin, 50 µg/mL streptomycin, 1 mM sodium pyruvate and 10% fetal bovine serum. Tau RD P301S FRET Biosensor cells were seeded (35,000 per well) on a clear 96-well flat-bottomed cell culture plate the day before experiment.

#### Immunodepletion of brain homogenates

Protein A Dynabeads (Invitrogen, #10001D, 16.7 µL, 0.5 mg per well) were loaded onto a 96-well polypropylene PCR microplate (Axygen, #PCR-96M2). DynaMag-96 Side Magnet (Invitrogen, #12331D) was used to collect the beads and the supernatant was removed. Antibody (3 µg, 100 µL) was added to the beads in PBS + 0.02% Tween^®^ 20, resuspended and mixed by alternating the side magnets once every minute for 15 min. Unbound antibody was removed, and beads washed once with 200 µL PBS + 0.02% Tween^®^ 20. After washing of beads, tau seed material (AD brain homogenate 200 µg/mL; L66 brain homogenate 5 µg/mL) was added to the beads and incubated as above for 15 min. After incubation, immunodepleted supernatant was transfected into Tau RD P301S FRET Biosensor cells using Lipofectamine 3000 Transfection Reagent as per manufacturer instructions (Invitrogen, #L3000008). Each condition was performed in duplicate.

#### Quantitative assessment of tau seeding *via* flow cytometry

Medium was removed from the test wells and cells were detached using 40 µL trypsin-EDTA (0.05%) solution. Trypsin was neutralised with 160 µL medium and samples transferred to a 96-well polypropylene V-bottom plate prior to centrifugation at 500 x g for 5 min. The supernatant was removed, and cells suspended in 200 µL PBS + 0.25% (w/v) BSA and live cell flow cytometry was performed using a BD LSR Fortessa Flow Cytometer.

A total of 20,000 cells were gated by forward scatter (area) vs. side scatter (area) bivariate plot. Single cells were subsequently defined by gating on a forward scatter (height) vs. forward scatter (area) bivariate plot. Cells were excited with a 405 nm laser with CFP measured with a 450/50 nm band width emission filter and YFP with a 525/50 nm band width emission filter. CFP vs. YFP bivariate plots were used to identify FRET-negative and positive cells with results displayed as a percentage. Data analysis was performed using FlowJo (10.7) software.

### Immunohistochemistry of human brain sections

Formalin-fixed paraffin-embedded human brain sections (5 μm) corresponding to Brodmann areas 20, 28 and 36 were received from South West Dementia Brain Bank under project code MTA147. These sections were from two age-matched donors, one donor with clinical and histological diagnosis of AD at Braak stage V/VI and a second donor which served as a healthy control (Braak stage I).

Primary antibodies used were those generated within this study. Positive control antibodies included HT7 (Fisher, #MN1000), a murine IgG1κ against tau epitope 159–163 AA, and AT8 (Invitrogen, #MN1020), a murine IgG1κ recognising a tau epitope phosphorylated at amino acids 202 and 205. Protein A-purified mouse IgG (Immunoreagents, #Mu-003-C.01) was used as a negative control antibody. Dilutions for each primary antibody used are displayed in the results.

Sections were dewaxed and immunohistochemistry performed using BOND Polymer Refine Detection Kit (Cat #: DS9800, Leica Biosystems, UK) using Leica BOND III instrument. Epitope retrieval 1 solution (Citrate buffer, #AR9961; Leica Biosystems) was incubated for 20 min at 100 °C then incubated with hydrogen peroxide for 10 min followed by washing with BOND wash solution (Leica, #AR9590) three times. Sections were then incubated for 1 h with primary antibody prepared in 3% BSA in PBS (pH 7.4). After washing with BOND wash solution, sections were incubated with secondary rabbit anti-mouse IgG (Cat #: DS9800, Leica Biosystems, UK) and polymer anti-rabbit Poly-HRP-IgG (Cat #: DS9800, Leica Biosystems, UK) each for 10 min. After washing, sections were incubated with DAB for 10 min followed by washing with deionized water. Sections were subsequently counterstained with haematoxylin, dehydrated in a graded ethanol series and then xylene before being cover slipped using a non-aqueous mounting media (Leica CVMount). Sections were scanned using Zeiss Axioscan Z1 slide scanner at 20x and imaged using Zeiss Axioscope 5 upright microscope at 20x magnification.

### Immunogold electron microscopy labelling of tau filaments isolated from AD brain tissue

Sarkosyl-insoluble samples containing tau filaments were isolated from AD patient frontal cortex as per previously published methods [50]. Filaments (3 µL) were placed onto the centre of a formvar/carbon-coated 400 mesh copper grid (EM Resolutions, # FC400Cu25) for 2 min. For all incubation steps, grids were placed on 25 µL drops on parafilm. The grids were blocked for 10 min in blocking buffer (0.5% fish skin gelatine in PBS). Blocking was followed by overnight incubation in 10 µg/mL primary antibody diluted in blocking buffer, at 4 °C. Grids were washed six times in blocking buffer, then incubated for 45 min in goat anti-mouse IgG conjugated 10-nm colloidal gold particles (Sigma, #G7652), diluted 1:25 in blocking buffer. Following incubation, the grids were washed six times with PBS, followed by a 5 min incubation on 2% glutaraldehyde in PBS. Fixation was followed by three 5 min washes in PBS and two 5 min washes in water. Finally, each sample was negatively stained by incubation on a drop of UranyLess EM Stain (Electron Microscopy Sciences, #22409) for 1 min. Samples were left to dry and then viewed under a JEOL 1400 plus transmission electron microscope with digital image capture at 25,000x magnification.

## Results

### Generation of high-affinity sheep anti-tau antibody panel against novel epitopes

Hyperimmunisation of two Welsh-bred sheep with either full-length tau protein (2N4R tau) or truncated tau (dGAE, representing the PHF-core domain of 2N4R tau corresponding to residues 297–391 first isolated from AD PHFs [43, 44]) generated antigen-specific immune responses. Two single chain variable fragment (scFv) libraries were constructed with the resultant antibody repertoire following published methods [11, 47]. The dGAE region was of particular interest, given that it is present in the Pronase-resistant PHF-core and because of its prominent role in the pathologic aggregation cascade [58, 60]. Library 1 (2N4R tau as immunogen) contained 1.2 × 10^9^ unique clones and library 2 (dGAE as immunogen) contained 1.6 × 10^9^ clones. Biopanning strategies were designed to drive the enrichment of high-affinity binders to the PHF-core region. In addition, Forced Epitope Selection (FES), using peptide antigens representing non-core regions of tau, were incorporated into biopanning rounds to identify high-affinity binders across the entirety of the tau protein. To illustrate the success of the selection strategies, 142 unique scFv sequences were isolated and expressed as soluble single chain antibodies (scAbs) in a bacterial expression system for further characterisation.

**Fig. 1:**
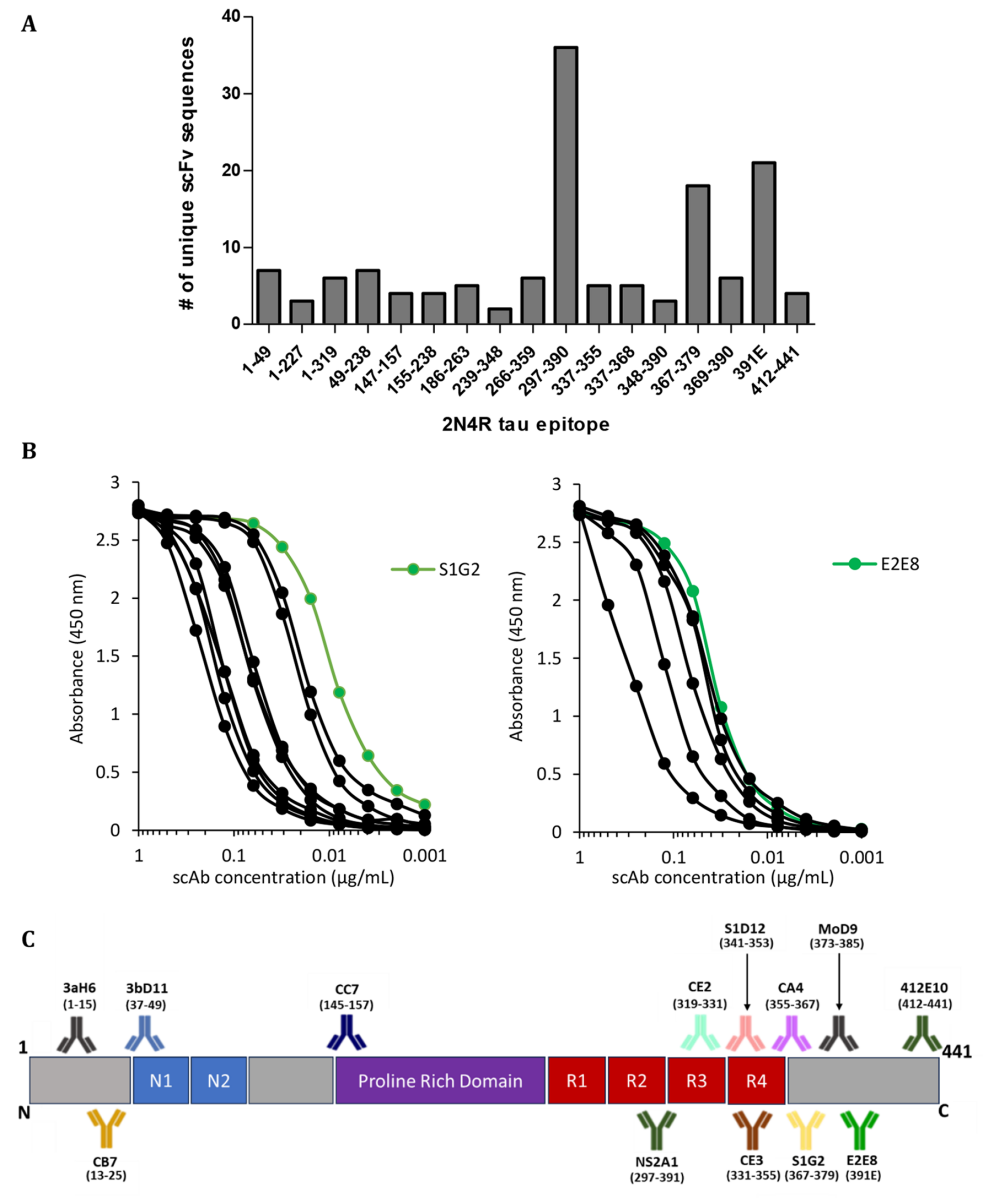
Summary of epitope diversity and antigen-binding characteristics of anti-tau antibodies generated from phage display libraries – (**A**) The scFv diversity generated through biopanning and their ability to bind regions along the length of the tau protein (2N4R tau). Number of unique clones representing various epitope regions of tau as determined by peptide ELISA. (**B**) Examples of relative immunoreactivity measurements of ten 367–379 AA specific scAbs binding to 2N4R tau (left panel) and six 391E specific scAbs binding to dGAE (right panel) in ELISA. The scAb with greatest immunoreactivity in each group was selected as the lead clone and is shown in green. (**C**) Schematic representation of the epitopes recognised by the final panel of lead scAbs. Differing colours of antibody are for presentation purposes only and to highlight the different epitope classes they bind.

A series of ranking protocols were employed to narrow down the number of scAbs to a panel of lead antibodies. One of the most effective was epitope recognition. Initially, epitopes were mapped to large tau fragments (100s AAs) then narrowed to medium-sized peptides (18–40 AAs) and finally to an overlapping library of 13 AA peptides that covered the entire tau protein (Fig. 1A). Where epitopes were recognised by multiple binders, affinity ranking was undertaken using ELISA (Fig. 1B) and subsequently, surface plasmon resonance (SPR), using immobilised 2N4R tau ligand as antigen. The binding affinity of scAbs ranged from high nanomolar (Clone MoD9, epitope region 373–385; *K*_D_ 452 nM) to sub-nanomolar (Clone S1D12, epitope region 341–353; *K*_D_ 0.52 nM) and their affinities largely correlated with their ranking using ELISA affinity data; the clone with greatest immunoreactivity from each epitope group was also the one having the lowest *K*_D_ value (see Supplementary Table S3 for full kinetics and affinity measurements).

Lead scAbs binding unique epitopes (Fig. 1C) were converted into sheep-mouse chimeric monoclonal IgGs and expressed in a transient HEK293 system. Conversion from scAb to mAb (monovalent to bivalent format) of the lead panel of antibodies resulted in an average 11.9-fold increase in binding affinities by SPR (Table 1 and Supplementary Table S4). Antibodies from this panel exhibit higher affinity against 2N4R tau when compared to a range of academic and commercially available antibodies (Tau12, Tau46, BT2, HT7, 7/51, 27/499 and mAb 423; Supplementary Table S5).

**Table 1:** Comparison of scAb and mAb equilibrium dissociation constants (K_D_) against 2N4R tau - performed using surface plasmon resonance of lead clones with immobilised 2N4R tau as described in Methods. E2E8 is not included in the table as it does not bind 2N4R. 3bD11 scAb was not tested as only scAb since it was not converted to mAb. N/A = not applicable

Clone (epitope)	scAb K_D_ (nM)	mAb K_D_ (nM)	Fold Improvement in K_D_ (nM)
3aH6 (1–15) 3bD11 (37–49) CB7 (13–25) CC7 (145–157) CE2 (319–331) S1D12 (341–353) CE3 (331–355) CA4 (355–367) S1G2 (367–379) NS2A1 (297–391) MoD9 (373–385) 412-E10 (412–441)	3.58 N/A 7.79 44.4 29.7 0.52 16.7 9.50 0.91 8.89 452 7.75	0.05 1.49 3.79 6.92 11.4 0.20 2.67 1.26 0.12 4.46 22.1 9.00	72 N/A 2.1 6.4 2.6 2.5 6.3 7.5 7.6 2.0 21 0.9

### Defining the minimal epitopes for selected core-tau antibodies

**Fig. 2:**
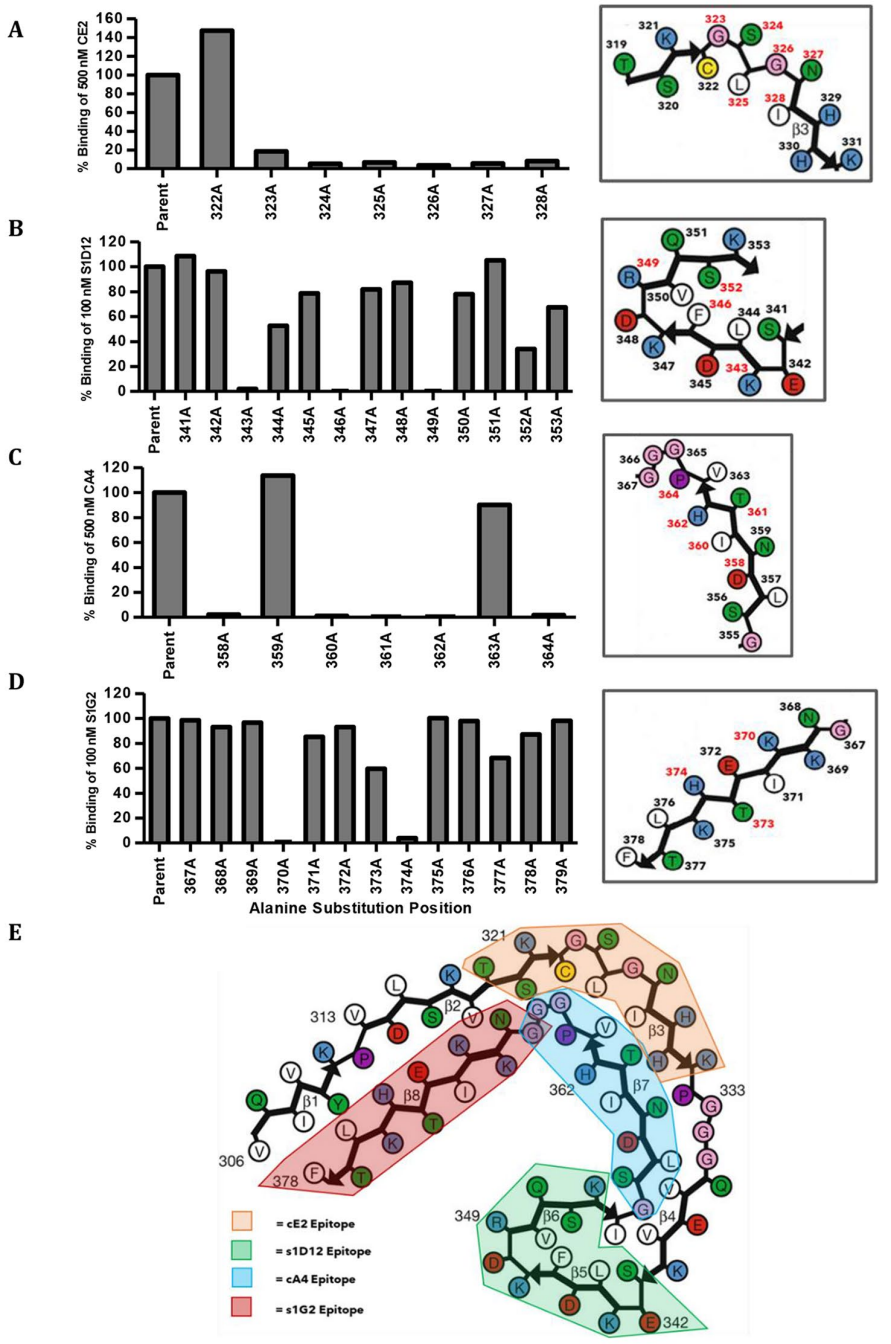
Determining key amino acids for antigen recognition of core-tau binders of interest - Percentage binding of CE2 (**A**) S1D12 (**B**) CA4 (**C**) S1G2 (**D**) scAbs to ASM peptides in relation to their respective parental peptide sequences. Critical amino acids for antibody binding are shown with red numbers in the inset image for each of the four clones (**E**) CE2, S1D12, CA4 and S1G2 epitopes in relation to published cryo-EM structure of AD tau filaments [21]. Amino acid colour coding: white - hydrophobic, blue - positive, red - negative, green - polar, pink - zwitterion, purple - proline, yellow - cysteine. Image modified from [21]

The use of a library of 13 AA peptides provided a starting point for characterising critical antigenic determinants. Alanine scanning mutagenesis (ASM) was adopted to identify specific residues required for the binding of four lead scAbs (CE2, S1D12, CA4 and S1G2), all with epitopes in the core region of pathologic filaments [21, 58]. Binding of CE2 to peptide 319–331 was completely lost by alanine substitution of any amino acid within the 323–328 region (Fig. 2A). This 6 AA epitope appears to be linear and contains two polar residues (S324 and N327) and two glycine residues (G323 and G326). Since glycine residues provide conformational flexibility, it seems likely that charge and flexibility in the epitope are important for CE2 binding. Similarly, ASM peptides for the region 341–353 revealed that amino acids at positions 343, 346, 349 and 352 were critical for S1D12 binding, defining a non-sequential epitope (Fig. 2B). This epitope has an overall positive charge and contains a high number of charged residues (4 positive amino acids (K343, K347, R349 and K352) and 3 negative residues (E342, D345 and D348)). For CA4, alanine substitution of AAs at positions 358, 360, 361, 362 and 364 prevented binding whereas changes at 359 and 363 had no effect (Fig. 2C). Binding of S1G2 was completely lost by a 370 or 374 substitution while a 373 substitution also greatly reduced immunoreactivity (Fig. 2D). S1G2 is predicted to bind a “pocket” of four positively charged amino acids (K369, K370, H374 and K375) which flank one negatively charged AA (E372) with K370 and H374 being the innermost positive residues and the critical AAs for this epitope pocket. To further verify these findings, S1G2 scAb was covalently bound to a CM5 chip and SPR measurements performed to assess the kinetics and affinity of binding of this scAb to different ASM peptides. SPR confirmed total abrogation of S1G2 binding when alanine substitutions were at positions 370 or 374, whereas the 373-substitution reduced binding affinity from 28.1 nM to 168 nM (6-fold, Supplementary Table S6).

### Developing sensitive diagnostic pairs for the interrogation of mixed tau species (“tauosome”)

**Fig. 3:**
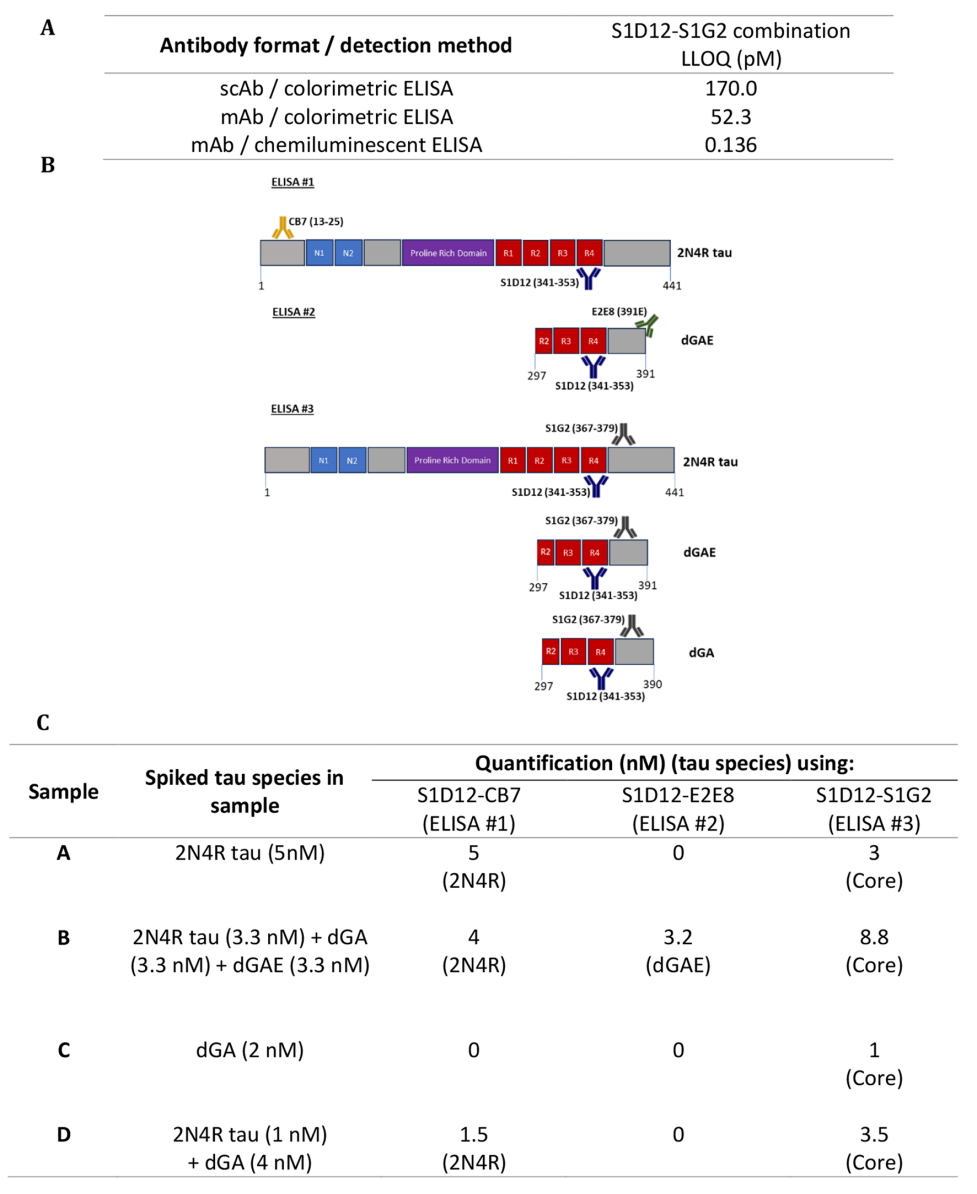
Sensitive paired ELISAs for the detection and quantification of core-containing tau species **– (A)** Sensitivity of SD12-S1G2 immunoassay using different ELISA detection methods showing that the chemiluminescent detection of HRP-conjugated mAb achieved the greatest sensitivity. **(B)** Schematic representation of 3 paired ELISAs capable of distinguishing mixed tau fragment samples. ELISA #1 identifies fragments containing AA residues 13–353 AAs of 2N4R tau. ELISA #2 only recognises fragments C-terminally truncated at Glu-391. ELISA #3 should identify any tau species containing amino acid residues 337–367 in the core region of 2N4R tau. **(C)** Summary of various tau species and their concentrations determined using paired ELISA. Concentrations of various tau fragments in the spiked sample mix were calculated using a standard curve and the identity of the spiked sample was deduced on the basis of the epitopes of the capture and detector antibodies used. A value of zero indicates no binding observed.

High-affinity tau antibodies were paired in various combinations in a sandwich ELISA format to determine the lower limits of quantification (LLOQ) for various tau species. The assay was initially optimised for 2N4R tau quantification using S1D12 as the most effective capture antibody and S1G2 as the most sensitive detector antibody, each having sub-nM affinities and epitopes within the core region (Table 1). A chemiluminescent ELISA improved the LLOQ from 52.3 pM to 136 fM, compared with the colorimetric ELISA, enabling greater sensitivity for the detection of core-containing tau fragments (Fig. 3A). All other combinations of tau antibody pairings are summarised in Supplementary Table S7 and the three best combinations were utilised to introduce the concept of the “tauosome” and that these novel antibody combinations could be used to detect unexplored tau fragments and a more complete interrogation of tau (Fig. 3B).

In order to introduce this concept, the ability of these assays to detect different tau fragments in mixtures was investigated by spiking tau fragments at various concentrations and combinations. S1D12 was used as the universal capture mAb; the concentrations of various spiked species were calculated, and the identity of these tau fragments deduced (Fig. 3C). In ELISA#1, only samples A, B and D were detected using the antibody pair S1D12-CB7, as the epitope for CB7 detector (13–25 AA) is not present within dGA (297–390 AA). By simply not detecting Sample C in this assay, the absence of any N-terminal region containing fragment was confirmed. Simultaneously, based on the absorbance values of unknown spiked samples, the concentration of fragments possessing the tau N-terminal domain was calculated from the standard curves.

### Importance of antibody affinity as a factor for tau aggregation inhibition potency

**Fig. 4:**
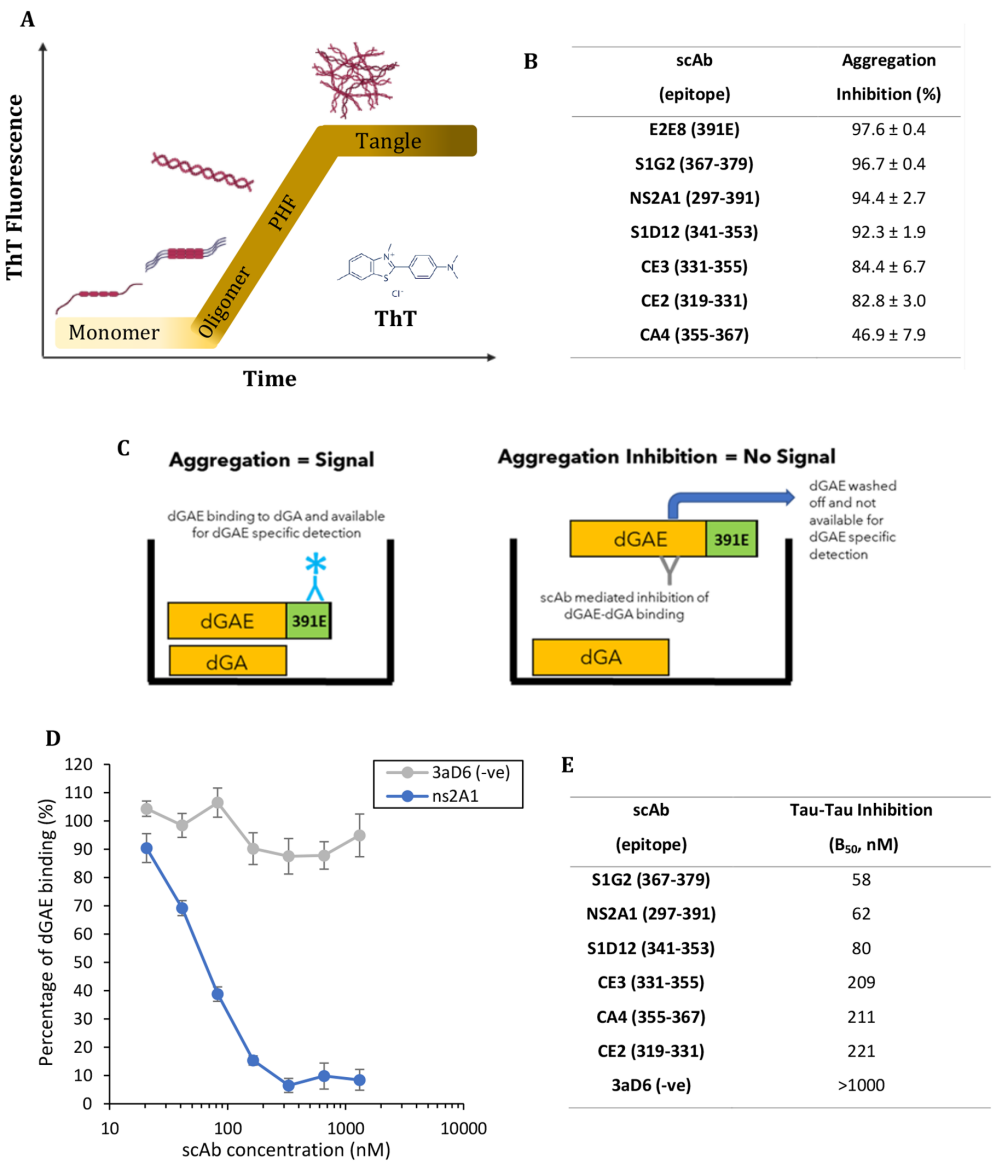
Characterisation of dGAE aggregation inhibition properties of the anti-tau scAb panel - (**A**) Schematic representation of the thioflavin T assay showing increased fluorescence with the progression of aggregation. (**B**) Ranking the aggregation inhibition potency of lead core-region scAbs using the thioflavin T assay, where aggregation inhibition of dGAE was quantified by calculating the percentage change of peak fluorescence. One-way ANOVA with Dunnett’s test compared individual anti-tau scAbs to negative control (mean of six different negative control scAbs). (**C**) Schematic representation of tau-tau immunoassay (B_50_) used to rank anti-tau scAb aggregation inhibition (adapted from [51]). (**D**) An example of B_50_ assay, comparing NS2A1 with N-terminal -ve control 3aD6 scAb. *n* = 4 per scAb with six different -ve control scAbs (C- or N-terminal targeting) were used. (**E**) Table shows calculated B_50_ values for core scAbs.

The ability of core-tau antibodies to prevent tau aggregation was investigated using dGAE. Aggregation was monitored using thioflavin T (ThT), a fluorescent probe commonly used to monitor in vitro fibril formation (Fig. 4A). Tau scAbs were ranked *via* the extent of dGAE aggregation (100 µM, 24 h, 700 rpm, 37 °C) in the presence of 10 µM scAb panel and compared to dGAE aggregated in the presence of a negative control scAb (Fig. 4B).

Results show almost complete inhibition of aggregation for S1G2 (96.7 ± 0.37%, *P* < 0.001), E2E8 (97.6 ± 0.38%, *P* < 0.001), NS2A1 (94.4 ± 2.70%, *P* < 0.001) and S1D12 (92.3 ± 1.87%, *P* < 0.001). Strong aggregation inhibition properties were also seen with CE2 (82.8 ± 3.03%, *P* < 0.001), CE3 (84.4 ± 6.69%, *P* < 0.001) and less potent, but still statistically significant, with CA4 (46.9 ± 7.89% *P* < 0.05).

To verify these findings, a second immunoassay-based ranking was adapted from a method used to assess the inhibition of tau-tau binding by low molecular weight compounds [52]. The ability of the scAb panel to prevent dGAE (297–391 AA) binding to dGA (297–390 AA) was measured *via* specific detection of truncation at Glu-391, using mAb 423 (Fig. 4C) and the concentration of scAb that prevented 50% dGAE-dGA binding (B_50_) was calculated (Fig. 4D). Strong aggregation inhibition was observed for S1G2 (58 nM), NS2A1 (62 nM) and S1D12 (80 nM), whereas CE3 (209 nM), CE2 (211 nM) and CA4 (221 nM) revealed a modest ability to inhibit aggregation. The overall ranking was similar to the ThT assay (Fig. 4E and B) with the negative control scAb showing no inhibition.

**Fig. 5:**
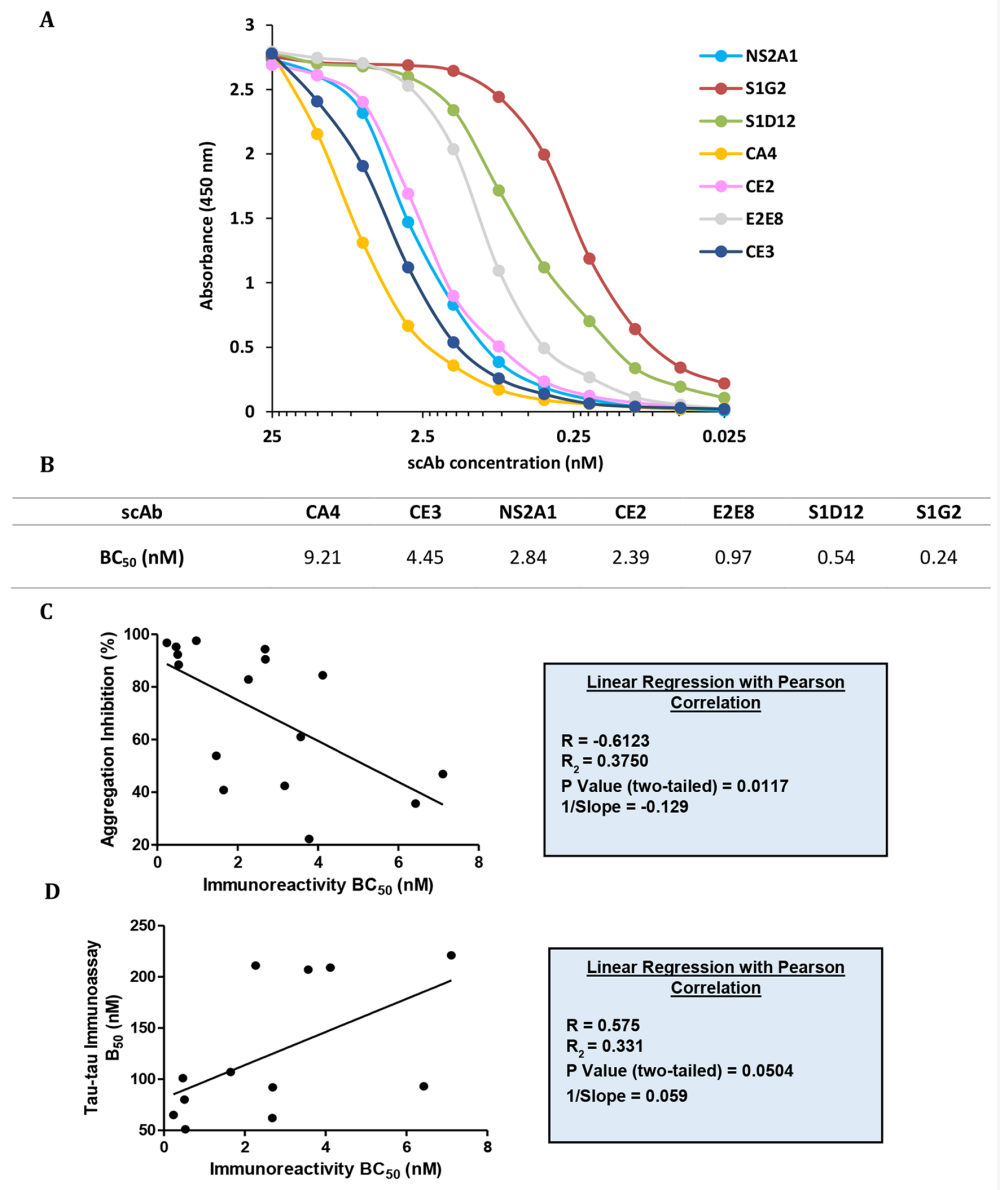
Correlation of antibody affinity and anti-aggregation potency – (**A**) Ranking the relative affinity of core binders against dGAE where a binding curve based on the immunoreactivity of scAbs was generated using average absorbance readings from 3 independent experiments. (**B**) BC_50_ values were calculated for each scAb via a 4-parameter logistic-regression model. (**C**) Linear regression and Pearson correlation of scAb immunoreactivity against aggregation inhibition properties in thioflavin-T assay. A strong negative correlation confirms that higher affinity scAbs have greater ability to inhibit aggregation. The correlation analysis includes all scAbs tested (*n* = 12); not just those in the lead panel. (**D**) Linear regression and Pearson correlation of scAb immunoreactivity against tau-tau immunoassay. A positive correlation confirms that higher affinity scAbs result in stronger inhibition of tau-tau aggregation. Correlation includes all scAbs tested (*n* = 16)

ScAb affinity for dGAE was ranked *via* ELISA, by calculating 50% bound (BC_50_) for each graph curve, using a 4-parameter logistic regression model (Fig. 5A). Results showed BC_50_ values for scAbs binding to dGAE ranged from 0.24 to 9.21 nM, with S1G2 showing the greatest binding to dGAE (0.24 nM), followed by S1D12 (0.54 nM), E2E8 (0.97 nM), CE2 (2.39 nM), NS2A1 (2.84 nM), CE3 (4.45 nM) and CA4 (9.21 nM) (Fig. 5B). Immunoreactivity to dGAE for all scAbs was plotted against their respective thioflavin T aggregation inhibition data and analysed by linear regression with a Pearson correlation. This revealed a significant inverse correlation between immunoreactivity as measured by BC_50_ and aggregation inhibition properties (*R* = -0.612, *P* = 0.0017), i.e. an antibody having a greater affinity to dGAE was a more potent aggregation inhibitor (Fig. 5C). Similarly, a positive correlation between scAb affinities and B_50_ values measuring dGAE-dGA binding inhibition was observed for the tau-tau immunoassay, however did not quite reach statistical significance (Fig. 5D) (*R* = 0.575, *P* = 0.0504).

### Epitope and affinity dependent prevention of pathological seeding of tau

**Fig. 6:**
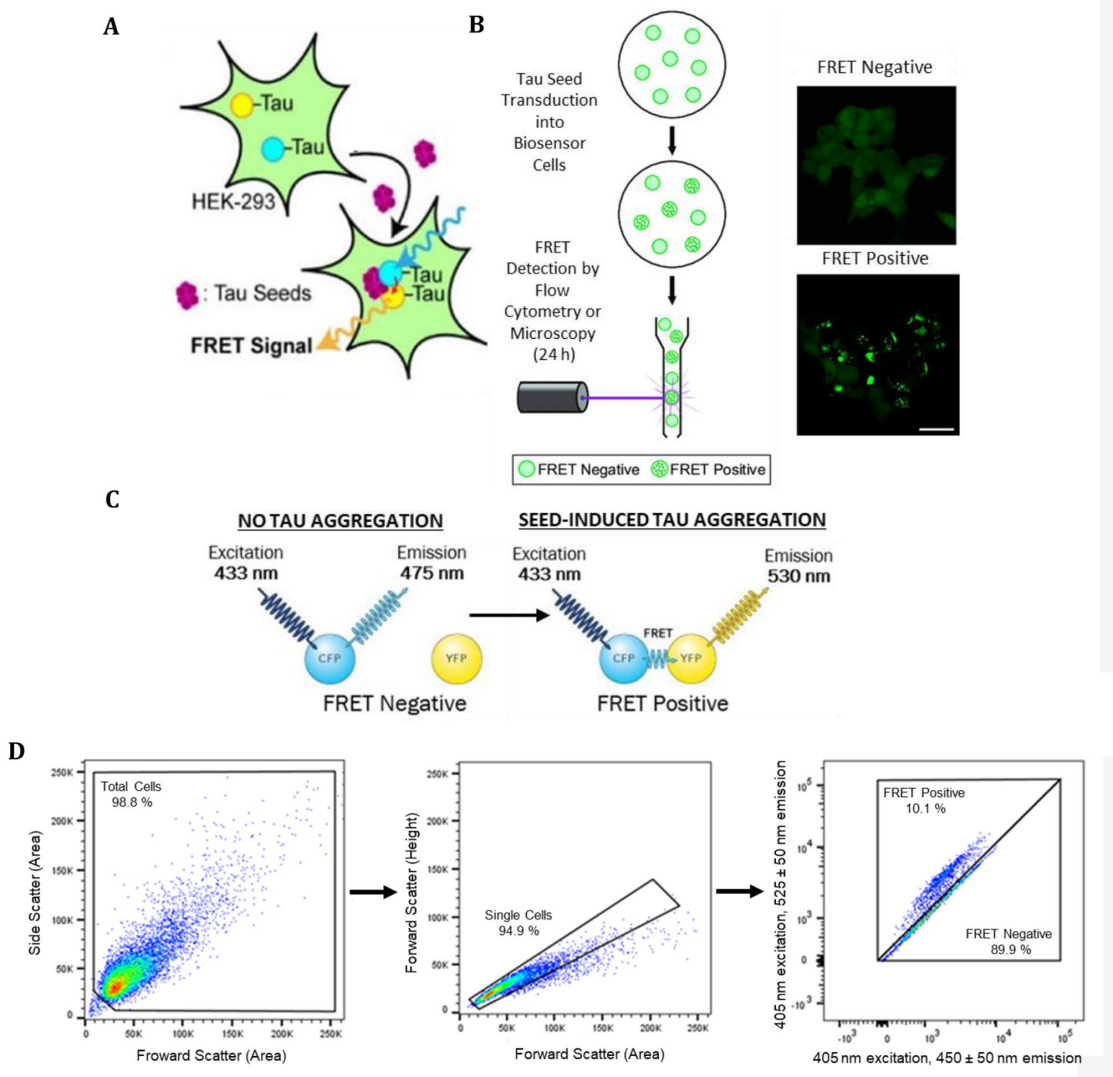
Schematic representation of tau seeding assay utilising Tau RD P301S FRET biosensor cells – (**A**) If tau seeds propagate into these cells and initiate aggregation of the two tau constructs, CFP can be excited with a laser causing YFP emission due to the energy transfer of these being in close proximity. (**B**) Seeding can be monitored as punctate fluorescence using the GFP channel of a microscope (Scale bar, 20 μm) or by quantitative flow cytometry by measuring FRET response on a CFP vs. YFP emission bivariate plot (**C**) When specifically exciting CFP, cells can be termed FRET negative if they emit at 475 nm bandwidth or FRET positive if emission is at 530 nm. Figure adapted from [16, 26]. (**D**) Representative gating strategy and data analysis of AD brain seeding Tau RD P301S FRET biosensor cells with FlowJo (10.7) software with further details provided within Materials and methods section

Therapeutic anti-tau antibodies are primarily aimed at inhibiting aggregated tau “seeds”, present in the extracellular space, from propagating between neurons in a prion-like manner. To evaluate the potential of our antibody panel to inhibit the propagation of aggregated tau, pathologic brain homogenate containing tau seeds were immunodepleted with anti-tau mAbs. The resulting supernatant was used to transfect HEK Tau RD P301S FRET biosensor cells and seeding measured *via* quantitative flow cytometry [26]; Fig. 6). This assay has been used previously to investigate tau seeding [18] and tau immunotherapy approaches [13, 26, 61]. Briefly, the uptake of tau seeds by the biosensor cells initiates aggregation of the two tau constructs, tau-CFP and tau-YFP, which results in a FRET response that can be measured using flow cytometry (Fig. 6). In this assay, AD brain homogenates and 5-month-old L66 brain homogenates (transgenic mice expressing 2N4R tau with P301S/G335D mutations specifically in neurons) were used as the source of tau seeds.

**Fig. 7:**
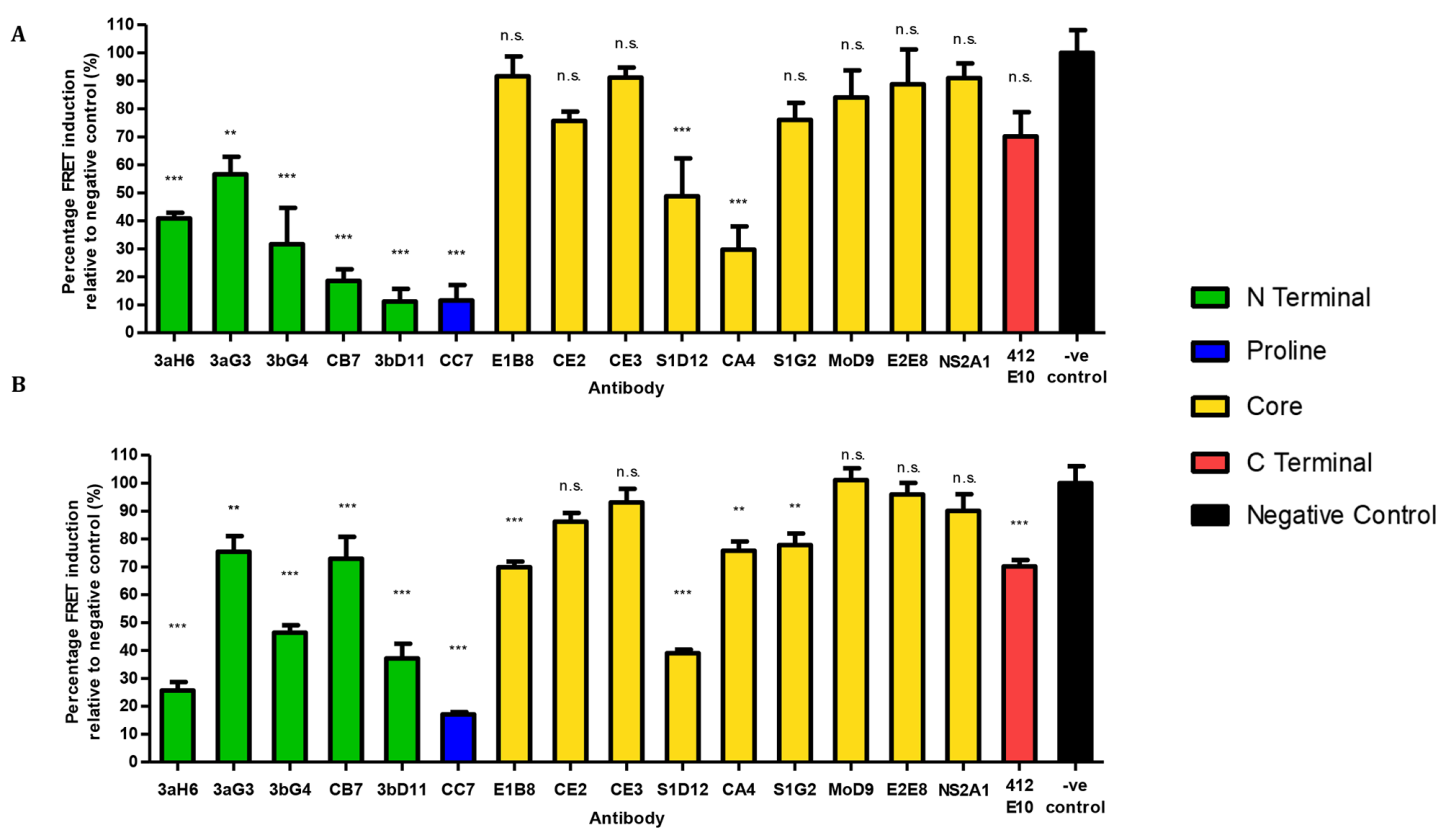
Antibody-mediated inhibition of brain homogenate seeding measured using flow cytometry - Percentage FRET signal generated in Tau RD P301S FRET biosensor cells transfected with (**A**) L66 brain homogenate (5 µg/mL) or (**B**) AD brain homogenate (100 µg/mL total protein) following immunodepletion with the tau mAb panel in comparison to a negative control antibody. The results are shown by the order of their epitopes along the tau protein (from N- to C-termini) and colour-coded to reflect the different protein domains as indicated. *N* = 4 (L66) or 5 (AD) performed in duplicate, values are expressed as mean ± SEM, one-way ANOVA was performed with Dunnett’s post hoc test to compare the negative control antibody (B11, which targets non-specific protein) with anti-tau antibodies, n.s. = not significant, *P ≤ 0.05, **P ≤ 0.01, ***P ≤ 0.001

Anti-tau mAbs were ranked by their ability to inhibit seeding of 5 µg/mL L66 brain homogenate when compared with a negative control mAb (Fig. 7A). A lower FRET signal in the assay, therefore, signifies superior mAb performance. All N-terminal binding mAbs tested showed statistically significant inhibition of L66 brain homogenate seeding ranging from 56.6 ± 6.2% (3aG3, *P* < 0.01) to 11.2 ± 4.6% (3bD11, *P* < 0.001) of FRET signal relative to the negative control. The proline region mAb CC7 showed potent inhibition (11.5 ± 5.6% compared to control; *P* < 0.001). None of the core or C-terminal binding mAbs showed statistically significant inhibition, with the exception of CA4 and S1D12 mAbs with both showing significant inhibition of FRET signal (29.7 ± 8.3%; *P* < 0.001 and 48.8 ± 13.6%; *P* < 0.001, respectively).

### Immunohistochemical staining of tau pathology in AD brain tissue

**Fig. 8:**
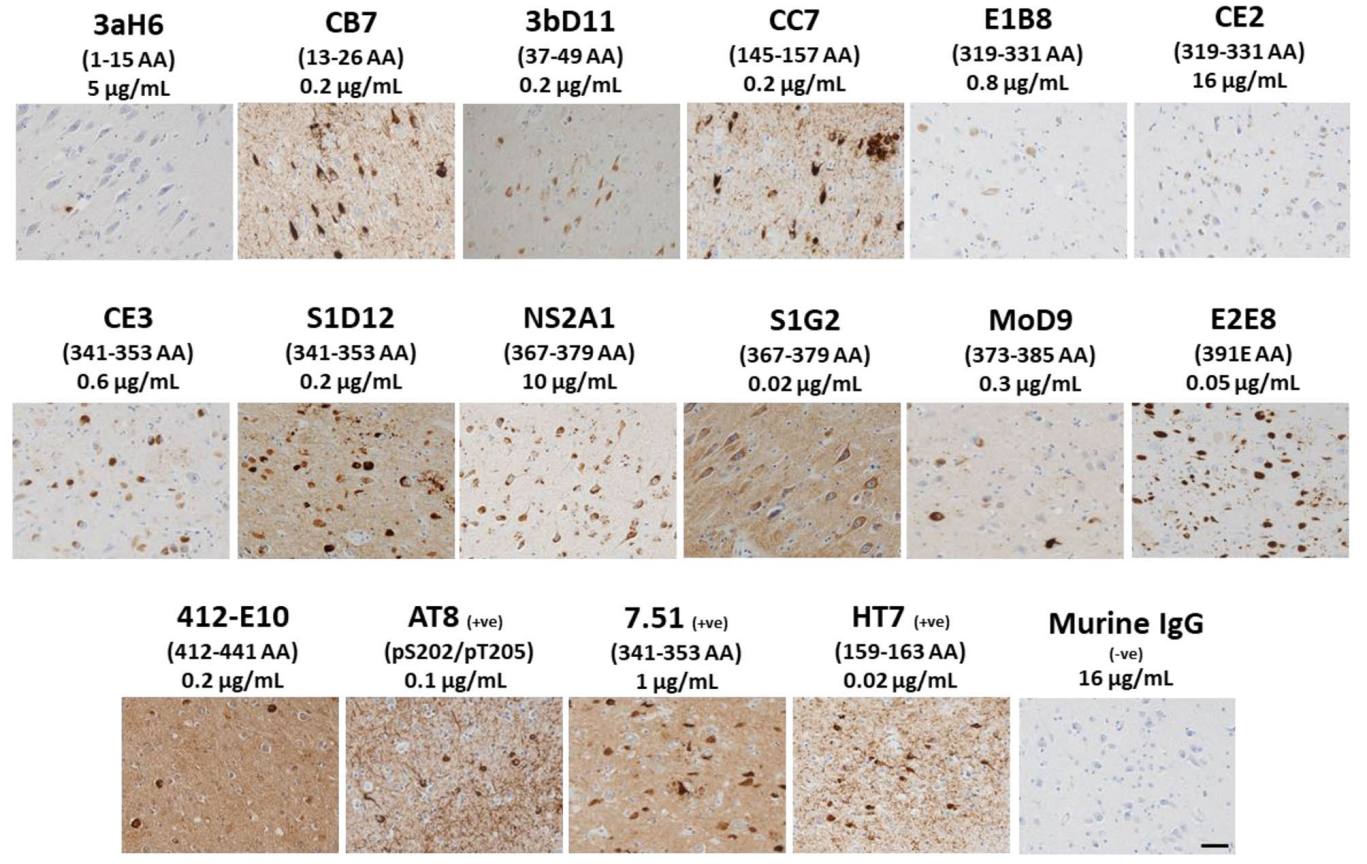
Immunohistochemical analysis of AD brain with anti-tau antibody panel - Paraffin-embedded AD brain sections of the frontal cortex (Brodmann areas 20, 28 and 36) were stained with antibodies as described and counterstained with haematoxylin. Images were taken using Zeiss Axioscope 5 at 20X magnification. Epitopes are indicated in parentheses with concentration of antibody as shown; +ve = positive control antibodies; -ve = negative control (protein A purified mouse IgG isotype control). Scale bar, 50 μm

There were subtle differences in immunohistochemical staining patterns of AD brain sections for the different antibodies and a more detailed charaterisation will be presented separately. Our anti-tau antibody panel recognised neurofibrillary tangles in AD brain sections of the frontal cortex, with a murine IgG isotype control showing no binding (Fig. 8). Most importantly, these antibodies showed no cross reactivity in sections from healthy controls (HC) (Figure S1). This is with the exception of NS2A1 (297–391 AA epitope) which showed comparable neuronal staining in sections from both HC and AD subjects and 412-E10 (412–441 AA epitope) showing modest staining in HC. This suggests either non-specific or cross-reactive binding to unrelated epitopes. Interestingly, despite 3aH6 (1–15 AA epitope) having very high affinity to recombinant tau, it showed very limited staining of tau pathology even at relatively high concentrations tested (5 µg/mL). This may reflect truncation or conformational differences between this epitope in full-length recombinant tau and in pathologic tau in situ.

### Immunogold staining reveals occluded and truncated epitopes in AD brain-derived tau filaments

**Fig. 9:**
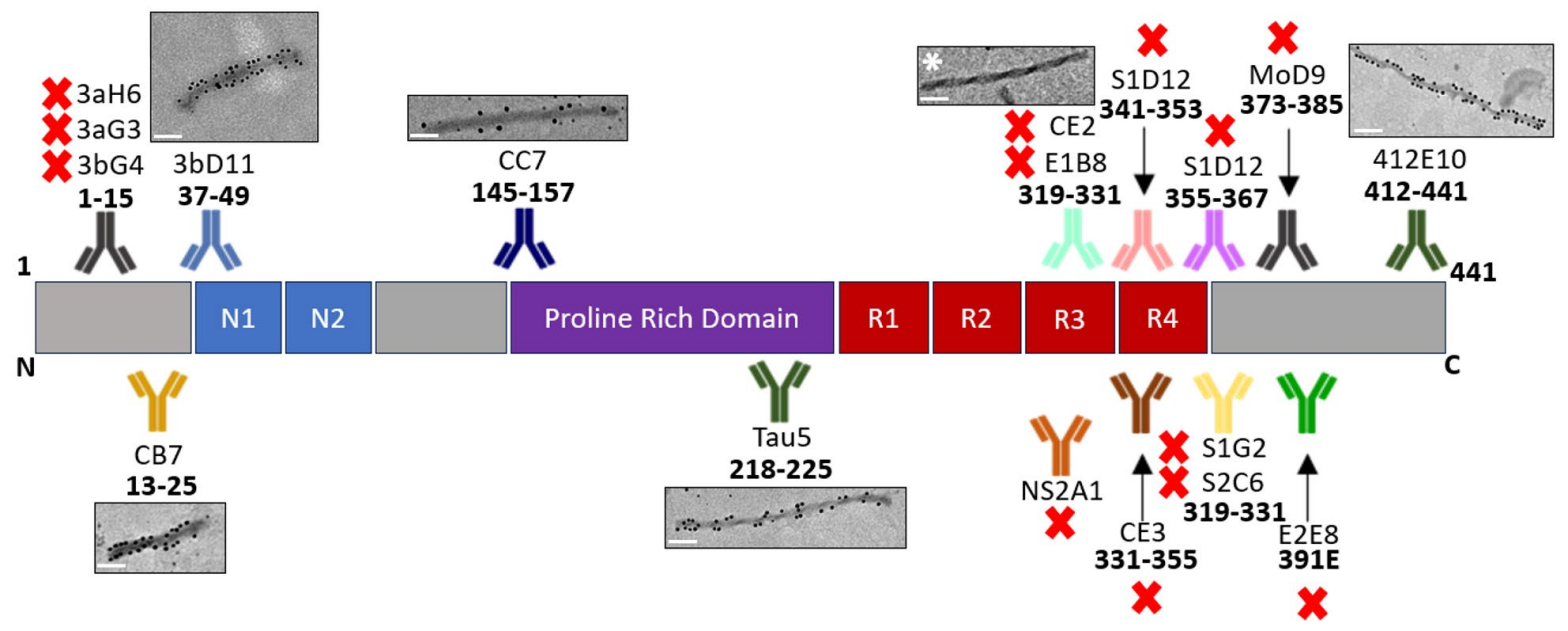
Immunogold staining of tau filaments derived from human AD brain using the anti-tau antibody panel; Tau5 antibody was used as a positive control. PHFs with no antibody binding are indicated by a red cross with mAb CE2 binding indicated by an asterisk provided as an example. Images taken at 25,000X magnification using TEM JEOL 1400 plus. Scale bar, 100 nm

Sarkosyl-insoluble tau filaments isolated from AD patient frontal cortex were visualised by transmission electron microscopy (TEM) and immunogold labelling of the tau mAb panel generated in this study. Extensive immunogold labelling was noted for N-terminal antibodies CB7 and 3bD11; proline region antibody CC7; and C-terminal region antibody 412-E10. No binding was detected when antibodies targeting the filament core were utilised (Fig. 9). This lack of signal suggests that epitopes between residues 319 to 385 and the 391E truncation site are occluded in the AD tau filament core whereas epitopes with positive binding likely contribute to the fuzzy coat of filaments. Interestingly, antibodies to residues 1–15 did not show any binding which may be indicative of a conformational change of the epitope or truncation mirroring the results from immunohistochemistry experiments (Fig. 8).

## Discussion

By adopting a sheep immunisation approach and phage display technology based stringent biopanning strategies, we have been able to generate a comprehensive panel of several high-affinity antibodies specific for regions spanning the full-length of tau protein. We have used two recombinant antibody libraries to select a panel of high-affinity binders recognising diverse epitopes. With the dGAE library, we specifically focused within the underserved core region of tau whereas the second full-length tau library was used to select antibodies recognising epitopes within the N terminus, C terminus and proline region of tau protein.

Using these antibodies binding different tau epitopes and pairing them as capture and detector antibodies, we were able to distinguish fragments representing different regions of tau in an ultrasensitive immunoassay format. This early evidence suggests the utility of a series of high-affinity mAb pairings to interrogate the “tauosome” in ways that have not been considered before. There is no clear evidence how a tau fingerprint might be used to aid in disease diagnosis, however the variety of antibodies developed in this study should prove informative. These antibodies will need to be investigated further with CSF and plasma samples to assess whether such antibody pairs can differentiate AD patients from healthy individuals based on their tau fingerprint. We believe this approach has real diagnostic potential beyond simpler studies measuring “total-tau” since tau exists as a complex milieu of fragments with differing truncations in health and disease [51]. The panel of high-affinity antibodies, having broad epitope diversity, could therefore help in the discovery of hidden fragments of tau with potential for diagnostic utility in different biological fluids.

Tau immunotherapy offers the potential to halt the cell to cell spread of pathologic core tau species. Therefore, we next assessed the aggregation inhibition properties of the dGAE antibody panel using a tau-tau binding immunoassay and a thioflavin-T assay and correlated the measurements with antibody affinities. Antibodies showing high affinity binding to dGAE exhibited the most potent inhibition of tau aggregation (Fig. 5). Our primary focus was the dGAE fragment, which represents part of the microtubule-binding region of tau found in the PHF core, because of its propensity to aggregate and drive the spread of pathology [2, 34, 58]. It has been shown previously that a core antibody exhibits superior inhibition of tau aggregation when compared with other antibodies targeting regions such as the N-terminus [3].

Thioflavin T data showed significant inhibition of aggregation using a scAb to dGAE molar ratio of 0.1:1 (Fig. 4B). Several factors support this substoichiometric scAb to dGAE ratio, such as: (i) dGAE may not be in a completely monomeric state, i.e. one antibody could therefore neutralise more than a single dGAE molecule; and (ii) tau aggregation is dependent on high concentrations of tau [2] and therefore binding of a small proportion of available dGAE may significantly impact aggregation time or the amount that can aggregate. The consideration that core-targeting antibodies could neutralise the aggregation propensity of multiple tau molecules concurrently is advantageous for tau immunotherapy and not widely considered in studies to date.

Conversion of scAbs to mAbs resulted in a 11.9-fold improvement in affinity on average, likely due to an increase in avidity (Table 1). The therapeutic potential of mAbs was ranked based on their ability to deplete pathological tau from brain homogenates and preventing ‘seeding’ into healthy cells (Fig. 7). The mAb CC7 (epitope 145–157 AA), shown as best in this assay, binds the proline region that has been considered therapeutically attractive by other groups [13, 42]. Although some of our N-terminal binders also displayed a modest degree of efficacy, generally antibodies recognising this region of tau have failed to show efficacy in clinical trials to date [40]. In an in vivo setting, high levels of disease-irrelevant N-terminal fragments present in CSF and blood can sequester antibodies targeting epitopes in this region and indirectly prevent them from binding to the pathologic seeding species as demonstrated in mouse and man [15, 51].

By developing a diverse panel of core-binding mAbs (319–379 AA) we were able to test the relative significance of affinity and epitope when inhibiting tau aggregation and seeding. CE3, which recognises a similar core epitope (epitope 331–355 AA) with a weaker affinity than S1D12 (epitope 341–353 AA), showed limited inhibition of tau seeding (10%), whereas the latter achieved seeding inhibition by approximately 60% (Fig. 7). The significance of target affinity has been demonstrated previously in a study where affinity maturation of the antibody fragment CBTAU-27.1 (epitope 299–318 AA) from 650 nM to 13 nM, resulted in a modest improvement in the ability of the antibody to inhibit tau seeding [3].

Targeting the correct epitope within the core also appears to be important, since mAb S1G2 (epitope 367–379 AA) showed limited ability to deplete tau seeds despite having a high affinity comparable with that of the potent inhibitor and core-tau binder, S1D12 (Fig. 7).

In general, similar levels and rankings of inhibition were observed in the FRET assay with the antibodies when homogenates from L66 mice or AD brains were used as tau seeds. However, one important observation was the consistently higher percentage of inhibition achieved by our antibody panel using homogenate from L66 brain compared to AD brain. All N-terminal and proline antibodies tested were human specific and therefore do not bind mouse tau (data not shown); this gives these antibodies a bias in favour of the human tau overexpressed in the L66 model and may explain the results observed. The most notable difference between L66 and AD brains was seen for the core-tau mAb CA4. For L66, CA4 was a potent inhibitor of tau seeding whereas for AD brain, CA4 achieved more modest, albeit statistically significant, inhibition. This may result from differences in the structure of tau filaments between the L66 model and AD, a hypothesis supported by a recent publication highlighting the striking diversity in tau filament structures across tauopathies [54]. L66 mice carry a P301S mutation that is associated with FTD [7] whose filaments differ in structure from that in AD [19]. This serves as a reminder that these core antibodies offer additional utility to aid our understanding of how and when these disease-critical epitopes may be occluded in different tauopathies.

Combining our findings on aggregation and seeding inhibition with insights from other studies [49, 62] strengthens the notion that having the optimal affinity for the optimal epitope could be crucial for effective therapeutic intervention *via* inhibition of tau aggregation and/or tau seeding. To date, the significance of antibody affinity has not received as much attention as the importance of the epitope recognised by potential therapeutic antibodies. Antibodies have been employed as therapeutics having a wide range of affinities, spanning from micromolar [49] to femtomolar [62]. In our present study, we have analysed the seeding potential using ex-vivo brain homogenates but further verification in the matrices of cerebrospinal or interstitial fluid is required.

EM analysis showed that none of our core-tau antibodies bound to PHFs derived from human AD brain in agreement with other studies that suggest the core region is occluded in these structures [3, 44]. This evidence is further supported by other methods utilising immunoassays with core-tau antibodies highlighting occlusion of core tau in PHFs [27, 31, 35]. Binding of core-tau antibodies to tau pathology by immunohistochemistry most likely reflects exposure of epitopes following fixation and then acidic and heat pretreatments of sections. Taken together, this confirms the presence of core tau in pathology and that the epitopes for this region are occluded in fully formed PHFs and tangles. Absence of mAb 3aH6 (epitope 1–15 AA) binding using EM and IHC is also of interest given the importance of truncation to the pathophysiology of tau in AD. However, this needs to be investigated further by IHC using freshly frozen tissues.

Considering the low levels of antibodies that enter (and/or exit) neuronal or other cell types [9], tau immunotherapy is likely to function extracellularly by binding to and preventing oligomeric tau seeding species from propagating into healthy neurons. The active, toxic species of tau is considered to consist predominantly of intermediate soluble oligomers with research supporting tangles as late-stage remnants of neurodegeneration [24, 41]. Future studies are necessary to investigate epitopes within the core and establish the stage at which occlusion occurs during the oligomerisation process. With S1D12 reducing tau seeding from the AD brain homogenate and CA4 doing the same from the L66 brain homogenate, and the fact that neither of these mAbs recognise PHFs suggests that these mAbs bind to early-stage oligomers rather than late-stage tangles. Since oligomers rather than tangles are responsible for anterograde trans-synaptic spreading and subsequent loss of brain function, particularly when found in synapses [12, 56], targeting such oligomers may have therapeutic significance.

## Conclusion

These well-characterised anti-tau antibodies, particularly those with picomolar affinity for the core-tau region present a valuable set of assets. They can serve as effective tools for advancing our understanding of the intricate nature of tau pathology and have the potential to aid in early diagnosis and treatment of AD and other tauopathies.

## Electronic supplementary material

Below is the link to the electronic supplementary material.

Supplementary Material 1

## Data Availability

All data are provided within the manuscript or the Supplementary figures/tables and the Supporting Information. Materials are available on reasonable request.

## Abbreviations

AD: Alzheimer’s Disease
ASM: Alanine Scanning Mutagenesis
BSA: Bovine Serum Albumin
cDNA: Complementary DNA
CFP: Cyan Fluorescent Protein
DAB: 3, 3’-Diaminobenzidine
dGA: 2N4R tau (297–390 AA) Fragment
dGAE: 2N4R tau (297–391 AA) Fragment
DMEM: Dulbecco’s Modified Eagle Medium
DNA: Deoxyribonucleic Acid
DTT: Dithiothreitol
EDTA: Ethylenediaminetetraacetic Acid
ELISA: Enzyme-Linked Immunosorbent Assay
EM: Electron Microscopy
FBS: Fetal Bovine Serum
FRET: Fluorescence Resonance Energy Transfer
FTD: Frontotemporal Dementia
HCl: Hydrochloric Acid
HEK: Human Embryonic Kidney
HRP: Horseradish Peroxidase
2N4R tau: Full-length tau (1-441 AA)
IgG: Immunoglobulin G
IHC: Immunohistochemistry
K_D_: Equilibrium Dissociation Constant
L66: Line 66
LLOQ: Lower Limit Of Quantitation
mAb: Monoclonal Antibody
MTBR: Microtubule-Binding Region
NFT: Neurofibrillary Tangles
PBL: Peripheral Blood Lymphocyte
PBS: Phosphate-Buffered Saline
PBST: Phosphate-Buffered Saline Plus Tween^®^ 20 (0.1%)
PEI: Polyethylenimine
PHF: Paired Helical Filament
PIPES: Piperazine-N, N′-bis(2-ethanesulfonic acid)
Ptau: Phosphorylated tau
RNA: Ribonucleic acid
scab: Single chain antibody
scFv: Single-chain variable Fragment
SDS-PAGE: Sodium Dodecyl-Sulfate Polyacrylamide Gel Electrophoresis
SPR: Surface Plasmon Resonance
TBS: Tris-Buffered Saline
TEM: Transmission Electron Microscopy
ThT: Thioflavin T
TMB: 3,3′,5,5′-Tetramethylbenzidine
VH: Variable Heavy
Vκ: Variable Kappa
Vλ: Variable Lambda
YFP: Yellow Fluorescent Protein
