# Supplementary material for: High-affinity antibodies specific to the core region of the tau protein exhibit diagnostic and therapeutic potential for Alzheimer’s disease
Source: Alzheimers Res Ther. 2024 Oct 2;16:209. doi: 10.1186/s13195-024-01561-1 (PMC11448309; doi:10.1186/s13195-024-01561-1)
Supplement: Supplementary file 1 — Supplementary Material 1 [file 13195_2024_1561_MOESM1_ESM.docx]

**Supplementary Material for:**

**High-affinity antibodies specific to the core region of the tau protein exhibit diagnostic and therapeutic potential for Alzheimer’s disease**

Mohammad Arastoo, Lewis K. Penny, Richard Lofthouse, Aya Abdallah, Anna Abrahamsson, Pietro Marini, Valeria Melis, Gernot Riedel, Charles R. Harrington, Claude M. Wischik, Andrew Porter and Soumya Palliyil

Alzheimer’s Research & Therapy

| **dGAE Library Selection** | **Pan 1** | **Pan 2** | **Pan 3** | **Number of positive clones**  **(and prefix for clone names)** |
| --- | --- | --- | --- | --- |
| **1** | 50 µg/ml dGAE | 10 µg/ml dGAE | 1 µg/ml dGAE | 5  (prefix-‘E’) |
| **2** | 100µg/ml dGA | 50 µg/ml dGA | 10 µg/ml dGA | 18  (prefix – ‘NS’ or ’Mo’) |
| **3** | 100 µg/ml dGA | 10 µg/ml dGA | 1 µg/ml dGA | 20  (prefix – ‘S’) |

**Table S1: dGAE library selection strategies -** *Three different selection strategies were used with the dGAE antibody library and the concentrations of dGA or dGAE antigen used for different rounds of panning are indicated. A total of 43 positive binders were isolated from the dGAE Library.*

| **2N4R tau Library Selection** | **Pan 1** | **Pan 2** | **Pan 3** | **Number of positive clones (and prefix for clone names)** |
| --- | --- | --- | --- | --- |
| **1** | 100 µg/ml 2N4R tau | 10 µg/ml 2N4R tau | 1 µg/ml 2N4R tau | 28  (prefix ‘C’) |
| **2** | 100 µg/ml R1-3 | 50 µg/ml R1-3 | 10 µg/ml R1-3 | 3  (prefix ‘C’) |
| **3** | 100 µg/ml 412-441 | 10 µg/ml 412-441 | 1 µg/ml 412-441 | 4  (prefix ‘412’) |
| **4** | 100 µg/ml 2N4R tau | 50 µg/ml 2N4R tau dGA deselection | 10 µg/ml 2N4R tau | 6  (prefix ‘3a’) |
| **5** | 100 µg/ml 2N4R tau | 10 µg/ml 2N4R tau dGA deselection | 0.1 µg/ml 2N4R tau | 2  (prefix ‘3b’) |

**Table S2: Full length tau library selection strategies -** *Five different selection strategies were used with the 2N4R tau library and the concentrations of 2N4R tau, R1-3 (amino acids 244-337) , 412-441 or dGA antigen used for different rounds of panning are shown. A total of 42 positive binders were identified from the 2N4R tau library.*

| **scAb (Epitope)** | ***K_a_* (1/Ms)** | ***K_d_* (1/s)** | **Chi^2^  (% of R_max_)** | ***K_D_* (nM)** |
| --- | --- | --- | --- | --- |
| **3aH6** (1-15) | 2.75 x 10^6^ | 9.87 x 10^-3^ | 3.5 | **3.58** |
| **CB7** (13-25) | 9.29 x 10^5^ | 7.23 x 10^-3^ | 7.2 | **7.79** |
| **CC7** (145-157) | 2.92 x 10^5^ | 1.29 x 10^-3^ | 1.6 | **44.4** |
| **CE2** (319-331) | 3.05 x 10^5^ | 9.07 x 10^-3^ | 2.3 | **29.7** |
| **S1D12** (341-353) | 8.49 x 10^5^ | 4.40 x 10^-4^ | 3.0 | **0.52** |
| **CE3** (331-355) | 1.04 x 10^5^ | 1.72 x 10^-3^ | 1.1 | **16.7** |
| **CA4** (355-367) | 8.12 x 10^4^ | 7.71 x 10^-3^ | 5.7 | **9.50** |
| **S1G2** (367-379) | 5.38 x 10^5^ | 4.82 x 10^-4^ | 1.1 | **0.91** |
| **NS2A1** (297-391) | 9.21 x 10^4^ | 7.23 x 10^-3^ | 4.2 | **8.89** |
| **MoD9** (373-385) | 1.42 x 10^5^ | 6.40 x 10^-2^ | 0.7 | **452** |
| **412-E10** (412-441) | 1.91 x 10^5^ | 1.49 x 10^-3^ | 3.0 | **7.75** |

**Table S3: Surface plasmon resonance-based kinetics and affinity measurements of scAb panel against 2N4R tau chip** *- Summary table of association rate constant (K_a_), dissociation rate constant (K_d_) and equilibrium dissociation constant (K_D_) of scAbs. Five concentrations (50, 25, 12.5, 6.25, 3.125 nM) of scAbs were flowed over a CM5 chip covalently bonded with 2N4R tau for 180 s before a 600 s dissociation phase. Chi^2^ is displayed as a percentage of maximal bound response units (R_max_) with lower values implying “goodness” of fit of the evaluation software model.*

| **mAb (Epitope)** | ***k_a_* (1/Ms)** | ***k_d_* (1/s)** | **Chi^2^  (% of R_max_)** | ***K_D_* (nM)** |
| --- | --- | --- | --- | --- |
| **3aH6** (1-15) | 3.13 x 10^6^ | 1.52 x 10^-4^ | 2.7 | 0.05 |
| **3aG3** (1-15) | 1.53 x 10^7^ | 4.75 x 10^-3^ | 3.6 | 0.31 |
| **CB7** (13-25) | 2.50 x 10^6^ | 9.47 x 10^-3^ | 3.5 | 3.79 |
| **3bD11** (37-49) | 2.67 x 10^6^ | 3.98 x 10^-3^ | 6.2 | 1.49 |
| **CC7** (145-157) | 4.58 x 10^6^ | 3.16 x 10^-2^ | 2.5 | 6.92 |
| **CE2** (319-331) | 4.58 x 10^5^ | 5.22 x 10-^3^ | 2.4 | 11.4 |
| **S1D12** (341-353) | 2.74 x 10^5^ | 5.43 x 10^-5^ | 0.2 | 0.20 |
| **CE3** (331-355) | 3.93 x 10^5^ | 1.05 x 10^-3^ | 2.7 | 2.67 |
| **CA4** (355-367) | 1.02 x 10^6^ | 1.29 x 10^-3^ | 1.2 | 1.26 |
| **S1G2** (367-379) | 3.57 x 10^5^ | 4.32 x 10^-5^ | 4.1 | 0.12 |
| **NS2A1** (297-391) | 2.43 x 10^5^ | 1.08 x 10^-3^ | 1.3 | 4.46 |
| **MoD9** (373-385) | 6.62 x 10^4^ | 1.46 x 10^-3^ | 0.1 | 22.1 |
| **412-E10** (412-441) | 1.75 x 10^6^ | 1.57 x 10^-2^ | 3.8 | 9.00 |

**Table S4: Kinetics and affinity measurements of the mAb panel against 2N4R tau chip using surface plasmon resonance -** *Summary table showing association rate constant (k_a_), dissociation rate constant (k_d_) and equilibrium dissociation constant (K_D_) of mAbs against 2N4R tau. Five concentrations (25, 12.5, 6.25, 3.125, 1.563 nM – highest to lowest on sensorgram) of mAb were flowed over an 2N4R tau CM5 chip. Association = 180 s, dissociation = 600 s. Chi^2^ is displayed as a percentage of maximal bound response units (R_max_) with lower values implying “goodness” of fit of the evaluation software model.*

| **mAb** | **2N4R tau BC50 (nM)** |
| --- | --- |
| S1D12 | 0.015 |
| 3aG3 | 0.019 |
| 3aH6 | 0.025 |
| 3bG4 | 0.029 |
| S1G2 | 0.032 |
| CA4 | 0.049 |
| 3bD11 | 0.066 |
| CB7 | 0.070 |
| Tau12 ***** | 0.074 |
| Tau46 ***** | 0.075 |
| CC7 | 0.083 |
| HT7 ***** | 0.122 |
| 412-E10 | 0.123 |
| MoD9 | 0.161 |
| BT2 ***** | 0.185 |
| 7/51 ***** | 0.369 |
| 27/499 ***** | 0.461 |
| CE3 | 1.654 |
| NS2A1 | 2.34 |
| CE2 | >10 |
| **mAb** | **dGAE BC_50_ (nM)** |
| E2E8 | 0.028 |
| 423* | 0.232 |

**Table S5: Immunoreactivity ranking of mAb panel and ‘gold standard’ academic/commercially available antibodies–** *Determined by mAb binding to 2N4R tau or dGAE via ELISA. All experiments were conducted independently five times, and the midpoint of the binding curve (BC_50_) was calculated using the average absorbance values. BC_50_ values were calculated using a 4-parameter nonlinear regression for each antibody against 2N4R tau and dGAE. * - academic/commercially available antibody.*

| **Peptide** | ***K*_D_ (nM)** |
| --- | --- |
| **Parent (367-379 AA)** | 28.1 |
| **367A** | 31.4 |
| **368A** | 36.1 |
| **369A** | 74.2 |
| **370A** | No binding |
| **371A** | 73.7 |
| **372A** | 53.1 |
| **373A** | 168 |
| **374A** | No binding |
| **375A** | 25.5 |
| **376A** | 17.1 |
| **377A** | 103 |
| **378A** | 23.3 |
| **379A** | 65.9 |

**Table S6: Binding profile S1G2 against N-terminally biotinylated ASM 13-mer peptides using Biacore X100 -** *ASM peptides (analyte) at five different concentrations (100, 50, 25, 12.5 and 6.25 nM) were flowed over a CM5 chip covalently bonded with S1G2 scAb. Association = 180 s, dissociation= 60 s.*

|  |  | **Detector mAb** | | | | | | | | |
| --- | --- | --- | --- | --- | --- | --- | --- | --- | --- | --- |
|  |  | **CB7** | **Tau12** | **CC7** | **HT7** | **BT2** | **S1D12** | **CA4** | **S1G2** | **Tau46** |
| **Capture mAb** | **CB7** |  | 174.4 | 21.8 | 21.8 | 21.8 | 174.4 | 87.2 | 21.8 | 43.6 |
|  | **Tau12** | 43.6 |  | 43.6 | 2.7 | 2.7 | 174.4 | 174.4 | 2.7 | 2.7 |
|  | **CC7** | No binding | | | | | | | | |
|  | **HT7** | 43.6 | 2.7 | 43.6 |  | 2.7 | 43.6 | 43.6 | 2.7 | 2.7 |
|  | **BT2** | 5.4 | 2.7 | 43.6 | 2.7 |  | 174.4 | 174.4 | 2.7 | 2.7 |
|  | **S1D12** | 174.4 | 10.9 | 174.4 | 10.9 | 5.4 |  | nb | 1.3 | 10.9 |
|  | **CA4** | No binding | | | | | | | | |
|  | **S1G2** | 21.8 | 2.7 | 174.4 | 21.8 | 1.3 | 43.6 | 21.8 |  | 5.4 |
|  | **Tau46** | 21.8 | 43.6 | 174.4 | 2.7 | 10.9 | 174.4 | 43.6 | 10.9 |  |

**Table S7: Combined LLOQs for optimised chemiluminescence diagnostic sandwich ELISAs** - *Values reported in pM. The S1D12/S1G2 and S1G2/BT2 pairings were most sensitive with an LLOQ of 1.3 pM. Values reported from individual ELISA plates with each pairing performed in triplicate. nb = no binding.*
